# Effects of aerobic exercise on pain and disability in patients with non-specific chronic low back pain: a systematic review protocol

**DOI:** 10.1186/s13643-019-1019-3

**Published:** 2019-04-22

**Authors:** Irlei dos Santos, Adriana Claudia Lunardi, Naiane Teixeira Bastos de Oliveira, Matheus Oliveira de Almeida, Leonardo Oliveira Pena Costa

**Affiliations:** Masters and Doctoral Programs in Physical Therapy, Universidade Cidade de São Paulo, Rua Cesario Galeno 448/475, Tatuapé, SP CEP 03071-000 Brazil

**Keywords:** Chronic low back pain, Aerobic exercise, Systematic review

## Abstract

**Introduction:**

Aerobic exercise programs have been used for various health conditions, including musculoskeletal disorders. However, the literature is still limited regarding the effect of aerobic exercise on pain and disability in patients with chronic non-specific low back pain.

**Methods:**

Search strategies will be performed in the following databases: PubMed, EMBASE (https://www.embase.com), CINAHL, PEDro, Lilacs, and Cochrane Central Register of Controlled Trials (CENTRAL). We will include randomized controlled trials in any language or date of publication. The primary outcomes will be pain and disability. The methodological quality and statistical reporting of each eligible trial will be evaluated using the 11-item PEDro scale. The strength of the recommendations will be summarized using the using the Grading of Recommendations Assessment, Development and Evaluation (GRADE) approach.

**Discussion:**

This systematic review will provide a synthesis of current evidence on the effects of aerobic exercise in patients with chronic low back pain on pain and disability outcomes. This information can help healthcare professionals in decision-making related to the use of aerobic exercise in patients with low back pain.

Following the guidelines, this systematic review protocol was registered on the Prospective International Register of Systematic Reviews (PROSPERO) number CRD42017071945.

**Electronic supplementary material:**

The online version of this article (10.1186/s13643-019-1019-3) contains supplementary material, which is available to authorized users.

## Strengths and limitations of the study

- This is a protocol of a systematic review of randomized controlled trials aimed to investigate the effects of aerobic exercise in patients with chronic low back pain on pain and disability outcomes.
- This review will follow all recommendations of the Cochrane Handbook of Systematic Reviews.
- The conclusions of this systematic review will be limited to the number and quality of the randomized controlled trials available.
- This systematic review will provide data that will aid in clinical decision-making for the use of aerobic exercise in patients with chronic low back pain. In addition, there is a potential for suggestions for new trials on this topic.

## Introduction

Low back pain is one of the most prevalent and disabling musculoskeletal conditions in the adult population and is considered a major public health problem worldwide [1, 2]. About 39% of the population will present complaints of low back pain at some point in their lives, being more frequent in females aged between 40 and 80 years old [1]. Low back pain represents one of the main causes of work absence and demand for healthcare, generating high costs for society [3]. In the USA, the direct and indirect costs of treating this symptom exceed 100 billion dollars per year, and more than 80% of all healthcare costs related to chronic conditions are spent on these patients [4].

Patients with chronic low back pain tend to seek treatment more often than patients with acute pain because the prognosis of these patients is not favorable [5]. The latest clinical practice guidelines recommend that patients remain physically active, as inactivity contributes negatively to recovery [6, 7]. Currently, it is known that general strength, conditioning, and resistance training programs for the spinal muscles, including aerobic exercises, are among the best treatment options for patients with chronic low back pain and have been shown to reduce pain and disability in the short and long term [8]. Aerobic exercise is defined as a form of exercise with relatively low intensity, with a duration ranging between 15 and 60 continuous minutes, and intensity of 60 to 90% of the maximum heart rate [9].

Aerobic exercise programs have shown physiological, psychological, and articular benefits in patients with chronic diseases (e.g., arthritis, osteoarthritis, and fibromyalgia) [10]. Chronic low back pain is associated with several changes in physical, emotional, and psychosocial dysfunctions that degrade quality of life [11]. Lack of physical conditioning and muscle disuse are common in these patients [11, 12]. Aerobic exercise stimulates the release of endorphins that relieve pain by inhibiting the pain pathways [13]. It also makes the patient more active, reducing the fear of moving (kinesiophobia) and increasing self-confidence [14]. Lastly, these exercises increase muscle blood flow and may reduce the stiffness commonly observed in patients with low back pain [15]. Finally, there is some evidence [16, 17] from observational studies showing that patients with low back pain can improve their symptoms by doing aerobic exercises such as running, cycling, or walking for example.

To date, only two systematic reviews have been published on the effectiveness of aerobic exercise in patients with low back pain [16, 18]. The first systematic review [16], published in 2015, verified the effect of aerobic exercise in patients with chronic low back pain and the authors concluded that aerobic exercises decrease pain, increase fitness, and improve psychological functioning [16]. Despite being the first systematic review with meta-analysis on aerobic exercise for patients with low back pain, this review has important limitations, i.e., the risk of bias of the included articles was not evaluated and all studies included were of observational design. These factors limit an in-depth evaluation of the effects of aerobic exercise on the treatment of patients with chronic low back pain [16].

The second systematic review published in 2016 aimed to review the effects of physical activity and exercise interventions (aerobic exercise, muscle strength and stabilization exercises, and/or flexibility training) in patients with chronic non-specific low back pain [18]. The authors concluded that aerobic exercise combined with other forms of therapy (flexibility, strength, and stabilization) would be beneficial in reducing the pain of patients with chronic low back pain [18]. However, this review also has some methodological limitations, i.e., it did not assess the risk of bias of the included studies and the sample included articles that compared non-aerobic exercise to other therapies or a control group [18].

In summary, neither reviews evaluated the isolated effect of aerobic exercise, adequately measured by randomized controlled trials [16, 18]. Thus, the objective of this systematic review will be to investigate the isolated effectiveness of aerobic exercise in patients with chronic non-specific low back pain measured in randomized controlled trials on pain and disability outcomes.

## Methods

This systematic review will be conducted following the guidelines of the Cochrane Handbook of Systematic Reviews [19]. We followed the Preferred Reporting Items for Systematic review and Meta-Analyses Protocols (PRISMA-P) (see Additional file 1) [19] guidelines and the checklist available (see Additional file 1).

### Patient and public involvement

There is no patient or public involvement on this protocol.

### Inclusion criteria of studies

#### Types of studies

This review will only include randomized controlled trials comparing the use of isolated aerobic exercise to any comparison group in patients with chronic non-specific low back pain. Non-randomized controlled trials will be excluded.

#### Participants

We will include studies that evaluated patients over 18 years of age and with chronic non-specific low back pain defined as pain or discomfort lasting more than 12 weeks in the region below the last costal margins and above the lower gluteal folds, with or without symptoms in the lower limbs [3]. If a study presents a mixed sample of patients with low back pain of different durations, we will request the separate data from the authors.

Studies will be excluded if they evaluated patients with nerve root compromise, metabolic or serious spinal pathologies (e.g., fractures, tumors, inflammatory, and infectious diseases), previous spinal surgery, postpartum low back pain or pelvic pain due to pregnancy, and pain unrelated to the lower back. In case separate data cannot be acquired, we will only include the articles with mixed population regarding duration and type of low back pain as long as most of the patients have chronic non-specific low back pain (> 75%).

#### Types of intervention and comparisons

The experimental intervention investigated in this systematic review will be aerobic exercise with a duration of 15 to 60 continuous minutes and intensity of 60 to 90% of the maximum heart rate [4]. We will include studies with aerobic exercise prescribed by any health professional. In addition, the main comparisons will be aerobic exercise versus placebo, aerobic exercise versus control without any intervention (e.g., waiting list), and aerobic exercise versus other interventions.

#### Assessed outcomes

The primary outcome assessed in this review will be pain intensity. The secondary outcomes will be disability, quality of life, return to work, and kinesiophobia measured by any validated instrument [20, 21]. Pain intensity, disability, and quality of life are the most important outcomes as per the most recent core outcome set for back pain patients [21].

### Study search and selection process

Searches will be conducted in the following databases: PubMed (https://www.ncbi.nlm.nih.gov/pubmed/), EMBASE (https://www.embase.com), CINAHL (https://www.ebscohost.com/), PEDro (https://www.pedro.org.au), Lilacs (http://lilacs.bvsalud.org/), and Cochrane Central Register of Controlled Trials (CENTRAL) (http://onlinelibrary.wiley.com/cochranelibrary/search?searchRow.searchOptions.searchProducts=clinicalTrialsDoi). Manual searches will also be carried out through the reference list of previous systematic reviews on the topic and of the clinical trials included in this review. We will also search for ongoing or unpublished studies by searching on the International Clinical Trial Registry Search Platform (http://apps.who.int/trialsearch/). Searches will not be restricted by language or date of publication [19, 22]. The search strategy was constructed by three main groups of terms related to (1) type of study [23], (2) chronic non-specific low back pain [23], and (3) aerobic exercise (Additional file 2). We will use all the existing synonyms for each search term (see Additional file 1). These terms are the same as those used by the Cochrane Back and Neck Review Group [23].

The studies will be assessed according to the eligibility criteria and the selection will be divided into two phases. The screening of titles, abstracts, and full texts will be screened by two independent reviewers. Any disagreement will be resolved by a third reviewer. If uncertainties persist as to the eligibility of an article, the authors may be contacted for clarification.

### Data extraction

A data extraction form will be used to extract data from each study. Data will be extracted on the size and characteristics of the sample (i.e., age, gender, duration of symptoms), characteristics of the interventions (number of sessions, duration of each session of treatment, intensity of training), instruments used to evaluate the outcomes, and results of the included studies. Two independent reviewers will perform the data extraction, and the disagreements will be resolved by a third reviewer. When data are not available in the manuscripts or in case of uncertainty, the authors may be contacted for clarification.

### Assessment of risk of bias

Risk of bias will be assessed with the PEDro (Physiotherapy Evidence Database) scale, which has good levels of validity and reliability and is strongly correlated with the Cochrane risk of bias tool [24, 25]. The PEDro scale evaluates the risk of bias and the statistical reporting of randomized controlled trials. This scale has 11 items: eight items (items 2–9) related to risk of bias (random allocation, concealed allocation, baseline comparability, blinded subjects, blinded therapists, blinded assessors, adequate follow-up, and intention-to-treat analysis) and two items (10 and 11) related to statistical reporting (between-group comparisons and point estimates and variability) [25]. The first item (eligibility criteria) is not considered in the total score because it is related to external validity.

The total PEDro score ranges from 0 to 10 points, and the higher the score is, the better the article in terms of risk of bias and statistical reporting [25]. For trials that are not available in the PEDro database, the scale will be applied by two independent reviewers and a third reviewer will mediate any disagreement [25]. Because blinding of therapists and patients in exercise trials are impossible, we will consider trials with a PEDro score of 8 as low risk of bias. Our primary analysis will include only trials with low risk of bias (i.e., 8/10). We will then conduct secondary analysis regardless the risk of bias.

### Measures of treatment effect

The treatment effects for continuous data will be reported as mean difference with 95% confidence intervals. If the outcomes are evaluated by different scales, we will calculate standardized mean differences and 95% confidence intervals. The treatment effects of categorical outcomes will be calculated using the risk ratio (RR) with 95% confidence intervals.

When there are sufficiently homogeneous clinical trials in a comparison, a meta-analysis will be performed using the random effects model for the follow-up periods: short term (outcomes assessed closer to 4 weeks post-randomization), medium term (outcomes assessed closer to 6 months after randomization), and long term (outcomes assessed closer to 1 year after randomization) [23]. In order to account for random errors due to small samples and multiple testing, we will use Trial Sequential Analysis [26]. For the interpretation of minimal clinically important difference from the patient’s point of view, we will consider 2 points on the Visual Analog Scale for Pain, ranging from 0 to 10, 5 points in the Roland Morris Disability Questionnaire, and 10 points in the Oswestry Disability Index in between-group comparisons.

### Heterogeneity analysis

The chi-square test will be used to identify the heterogeneity in the data from the studies. The magnitude of the heterogeneity will be confirmed by calculation of the *I*^2^ statistic (range from 0 to 100%) [25]. An *I*^2^ above 50% indicates significant heterogeneity and will result in a reduction of one level in the quality of the evidence due to inconsistency [23, 27].

### Synthesis of data

The quality of the evidence will be classified using the GRADE (Grading of Recommendations Assessment, Development and Evaluation) approach [27] and according to the Cochrane Handbook of Systematic Reviews [27]. Table 1 presents the GRADE approach [28]. As we expect some degree of heterogeneity, narrative synthesis of the results would be used as needed.

**Table 1:** Level of quality of evidence (GRADE)

Quality level	
High quality	
• There are consistent findings among at least 75% of participants. • Consistent low risk of bias, with accurate data and no known or suspected publication bias. • Future studies are unlikely to change the estimated results.	
Moderate quality	
• There is moderate confidence in the estimated effect. • Future studies are likely to have a significant impact on the confidence of the treatment effect estimation and may alter the treatment effect estimate.	
Low quality	
• Confidence in the treatment effect is limited. • Additional research is very likely to have a significant impact on the confidence of the estimated treatment effect and it is likely to change the estimate.	
Very low quality	
• Confidence in the estimated treatment effect is very limited. • Results are uncertain.	

The initial classification of the quality of the evidence is defined according to the design of the studies. The randomized controlled trial is the most appropriate study design for questions related to intervention and its quality of the evidence starts as high according to the GRADE approach [29]. The quality of the evidence will be based on five factors, where for each factor not found, the quality of the evidence may be downgraded by one level (from high to moderate, low, or very low). The five factors are:

- Methodological limitations (risk of bias): the quality of the evidence will be downgraded if there are methodological limitations that indicate a greater propensity for biases, thus reducing confidence in the estimation of the effect of the studies [29]. The evidence will be downgraded by one level when more than 25% of the studies included in a given comparison are classified as high risk of bias.
- Inconsistency: the quality of the evidence will be downgraded if significant heterogeneity is observed in the results, even after any sensitivity analysis of the hypotheses [30]. The evidence will be downgraded by one level when the inconsistency is greater than 50%.
- Indirect evidence: the quality of the evidence will be downgraded when participants, interventions, or outcomes from the assessed studies are essentially different from those considered in the research question or the clinical guideline or when there are no direct comparisons between the interventions [31]. The evidence will be downgraded by one level when more than 50% of the participants are not related to the target audience of the study.
- Imprecision: the main criterion used by the GRADE system to define the accuracy of the estimates is the 95% confidence interval [31]. The evidence will be downgraded by level when there are less than 400 participants in the comparison for continuous outcomes and fewer than 300 participants for categorical outcomes.
- Publication bias: the funnel plot [32] used for meta-analyses with ten studies or more will be used to verify publication bias. Studies with low accuracy and with small samples will be distributed symmetrically in the widest part of the funnel, and studies with higher accuracy and larger sample sizes will be closer to the actual result and located in the narrowest part of the funnel [32].

## Discussion

This systematic review aims to provide the best available evidence on the effectiveness of aerobic exercise in patients with chronic nonspecific low back pain on pain and disability outcomes. All recommendations of the Cochrane Handbook of Systematic Reviews will be followed for the review to be of high quality. We believe that the findings of this systematic review will be important because aerobic exercise is affordable, cost-effective, and commonly used by the general population. To date, we are not aware of any systematic reviews that have attempted to investigate the effects of aerobic exercise alone compared to other therapies in reducing the pain and disability of patients with chronic nonspecific low back pain. Therefore, this evidence will inform healthcare providers and patients about the potential benefits of this intervention. Furthermore, this review has the potential to identify gaps in the literature that could be addressed in future studies.

## Additional files

Additional file 1: PRISMA P - Preferred Reporting Items for Systematic review and Meta-Analyses Protocols. (PDF 568 kb)

Additional file 2: Description of data: detailed search strategy. (PDF 283 kb)

## Abbreviations

CENTRAL: Cochrane Central Register of Controlled Trials
EG: Example
GRADE: Grading of Recommendations Assessment, Development and Evaluation
PRISMA-P: Preferred Reporting Items for Systematic Review and Meta-Analyses Protocols
PROSPERO: Prospective International Register of Systematic Reviews
RR: Risk ratio
