# Supplementary material for: Effects of aerobic exercise on pain and disability in patients with non-specific chronic low back pain: a systematic review protocol
Source: Syst Rev. 2019 Apr 22;8:101. doi: 10.1186/s13643-019-1019-3 (PMC6477724; doi:10.1186/s13643-019-1019-3)
Supplement: Supplementary file 2 — Description of data: detailed search strategy. (PDF 283 kb) [file 13643_2019_1019_MOESM2_ESM.pdf]

Efficacy of aerobic exercise on pain and disability in patients with non-specific chronic low back pain: a systematic review protocol.

Authors: Irlei dos Santos[1], Naiane Teixeira Bastos de Oliveira[1], Matheus Oliveira de Almeida[1], Adriana Claudia Lunardi[1], Leonardo Oliveira Pena Costa[1].

1- Masters and Doctoral Programs in Physical Therapy - Universidade Cidade de São Paulo - São Paulo - Brazil.

E-mail: Irlei dos Santos - irlei_santos@yahoo.com.br; Naiane Teixeira Bastos de Oliveira – naiateixeira@hotmail.com; Matheus Oliveira de Almeida – mathewsalmeida@hotmail.com; Adriana Claudia Lunardi – adriana.lunardi@unicid.edu.br; Leonardo Oliveira Pena Costa – lcos3060@gmail.com.

Correspondence to:

Irlei dos Santos, FT, Ms
Universidade Cidade de São Paulo, Master’s and Doctoral Program in Physical Therapy
Rua Cesario Galeno 448/475, Tatuapé
São Paulo, SP
CEP 03071-000
Phone: 55 11 2178 1564
E-mail: mathewsalmeida@hotmail.com
Total manuscript words: 2446

Abstract

Introduction: Aerobic exercise programs have been used for various health conditions, including musculoskeletal disorders. However, the literature ~~is~~ still limited regarding the effect of aerobic exercise on pain and disability in patients with chronic non-specific low back pain.

Methods: Search strategies will be performed in the following databases: Pubmed, EMBASE (https://www.embase.com), CINAHL, PEDro, Lilacs, and Cochrane Central Register of Controlled Trials (CENTRAL). We will include randomized controlled trials in any language or date of publication. The primary outcomes will be pain and disability. The methodological quality and statistical reporting of each eligible trial will be evaluated using the 11-item PEDro scale. The strength of the recommendations will be summarized using the using the Grading of Recommendations Assessment, Development and Evaluation (GRADE) approach.

Discussion: This systematic review will provide a synthesis of current evidence on the efficacy of aerobic exercise in patients with chronic low back pain on pain and disability outcomes. This information can help healthcare professionals in decision making related to the use of aerobic exercise in patients with low back pain.

Following the guidelines, this systematic review protocol was registered on the Prospective International Register of Systematic Reviews (PROSPERO) number CRD42017071945.

Keywords: chronic low back pain, aerobic exercise, systematic review.

Strengths and limitations of the study:

- This is protocol of a systematic review of randomized controlled trials aimed to investigate the efficacy of aerobic exercise in patients with chronic low back pain on pain and disability outcomes.

- This review will follow all recommendations of the Cochrane Handbook of Systematic Reviews.

- The conclusions of this systematic review will be limited to the number and quality of the randomized controlled trials available.

- This systematic review will provide data that will aid in clinical decision making for the use of aerobic exercise in patients with chronic low back pain. In addition, there is the potential for suggestions for new trials on this topic.

1. INTRODUCTION

Low back pain is one of the most commonly prevalent and disabling musculoskeletal conditions in the adult population and is considered a major public health problem worldwide[1 2]. About 39% of the population will present complaints of low back pain at some point in their lives, being more frequent in females aged between 40 and 80 years old[1]. Low back pain represents one of the main causes of work absence and demand for healthcare, generating high costs for society[3]. In the United States, the direct and indirect costs of treating this symptom exceed 100 billion dollars per year, and more than 80% of all healthcare costs related to chronic conditions are spent on these patients[4].

Patients with chronic low back pain tend to seek treatment more often than patients with acute pain because the prognosis of these patients is not favorable[5]. The latest clinical practice guidelines recommend that patients remain physically active, as inactivity contributes negatively to recovery[6 7]. Currently, it is known that general strength, conditioning, and resistance training programs for the spinal muscles, including aerobic exercises, are among the best treatment options for patients with chronic low back pain and have been shown to reduce pain and disability in the short and long term[8]. Aerobic exercise is defined as a form of exercise with relatively low intensity, with a duration ranging between 15 and 60 continuous minutes, and intensity of 60% to 90% of the maximum heart rate[9].

Aerobic exercise programs have shown physiological, psychological, and articular benefits in patients with chronic diseases (e.g., arthritis, osteoarthritis, and fibromyalgia)[10]. Chronic low back pain is associated with several changes in physical, emotional, and psychosocial dysfunctions that degrade quality of life[11]. Lack of physical conditioning and muscle disuse are common in these patients[11 12]. . Aerobic exercise stimulates the release of

endorphins that relieve pain by inhibiting the pain pathways[13]. It also makes the patient more active, reducing the fear of moving (kinesiophobia) and increasing self-confidence[14]. Lastly, these exercises increase muscle blood flow and may reduce the stiffness commonly observed in patients with low back pain[15].

To date, only two systematic reviews have been published on the effectiveness of aerobic exercise in patients with low back pain[16 17]. The first systematic review[16], published in 2015, verified the effect of aerobic exercise in patients with chronic low back pain and the authors concluded that aerobic exercises decrease pain, increase fitness, and improve psychological functioning[16]. Despite being the first systematic review with meta-analysis on aerobic exercise for patients with low back pain, this review has important limitations, i.e., the risk of bias of the included articles was not evaluated and all studies included were of observational design. These factors limit an in-depth evaluation of the effects of aerobic exercise on the treatment of patients with chronic low back pain[16].

The second systematic review published in 2016 aimed to review the effects of physical activity and exercise interventions (aerobic exercise, muscle strength and stabilization exercises, and/or flexibility training) in patients with chronic non-specific low back pain[17]. The authors concluded that aerobic exercise combined with other forms of therapy (flexibility, strength, and stabilization) would be beneficial in reducing the pain of patients with chronic low back pain[17]. However, this review also has some methodological limitations, i.e., it did not assess the risk of bias of the included studies and the sample included articles that compared non-aerobic exercise to other therapies or a control group[17].

In summary, neither review evaluated the isolated effect of aerobic exercise, adequately measured by randomized controlled trials[16 17]. Thus, the objective of this systematic review will be to investigate the isolated effectiveness of aerobic exercise in patients with chronic non-

specific low back pain measured in randomized controlled trials on pain and disability outcomes.

2. METHODS:

This systematic review will be conducted following the guidelines of the Cochrane Handbook of Systematic Reviews[19]. We followed the Preferred Reporting Items for Systematic review and Meta-Analyses Protocols (PRISMA-P) (see Additional file 1)[18] guidelines and the checklist available (see additional file 1).

2.1 Patient and Public Involvement

There is no patient or public involvement on this protocol.

2.2 Inclusion Criteria of studies

2.2.1 Types of studies:

This review will only include randomized controlled trials comparing the use of isolated aerobic exercise to any comparison group in patients with chronic non-specific low back pain. Clinical trials that used inappropriate randomization processes will be excluded (e.g., alternated allocation, allocation by date of birth, etc.).

2.2.2 Participants

We will include studies that evaluated patients of both genders over 18 years of age and with chronic non-specific low back pain defined as: pain or discomfort lasting more than 12 weeks in the region below the last costal margins and above the lower gluteal folds, with or without symptoms in the lower limbs[3]. If a study presents a mixed sample of patients with low back pain of different durations, we will request the separate data from the authors.

Studies will be excluded if they evaluated patients with nerve root compromise, metabolic or serious spinal pathologies (e.g., fractures, tumors, inflammatory, and infectious

diseases), previous spinal surgery, postpartum low back pain or pelvic pain due to pregnancy, and pain unrelated to the lower back. In case separate data cannot be acquired, we will only include the articles with mixed population regarding duration and type of low back pain as long as most of the patients have chronic non-specific low back pain (> 75%).

2.2.3 Types of intervention and comparisons

The experimental intervention investigated in this systematic review will be aerobic exercise with duration of 15 to 60 continuous minutes and intensity of 60% to 90% of the maximum heart rate[4]. We will include studies with aerobic exercise prescribed by any health professional. In addition, the main comparisons will be: aerobic exercise versus placebo; aerobic exercise versus control without any intervention (e.g., waiting list), and aerobic exercise versus other interventions.

2.2.4 Assessed Outcomes

The primary outcomes assessed in this review will be pain intensity and disability as these outcomes are recommended by the core outcome set for back pain patients, measured by any validated instrument, and the secondary outcomes will be quality of life, return to work, and kinesiophobia[19 20].

2.3 Study search and selection process

Searches will be conducted in the following databases: Pubmed (https://www.ncbi.nlm.nih.gov/pubmed/), EMBASE (https://www.embase.com), CINAHL (https://www.ebscohost.com/), PEDro (https://www.pedro.org.au), Lilacs (http://lilacs.bvsalud.org/) and Cochrane Central Register of Controlled Trials (CENTRAL) (http://onlinelibrary.wiley.com/cochranelibrary/search?searchRow.searchOptions.searchProducts=clinicalTrialsDoi). Manual searches will also be carried out through the reference list of previous systematic reviews on the topic and of the clinical trials included in this review.

Searches will not be restricted by language or date of publication[18 21]. The search strategy was constructed by three main groups of terms related to 1) type of study[22] 2) chronic non-specific low back pain[22], and 3) aerobic exercise. We will use all the existing synonyms for each search term (see additional file 1). These terms are the same as those used by the Cochrane Back and Neck Review Group[22].

The studies will be assessed according to the eligibility criteria and the selection will be divided into two phases. Initially, the titles will be selected by one reviewer, then two independent reviewers will read the abstracts and full texts. Any disagreement will be resolved by a third reviewer. If uncertainties persist as to the eligibility of an article, the authors may be contacted for clarification.

2.4 Data Extraction

A data extraction form will be used to extract data from each study. Data will be extracted on the size and characteristics of the sample, characteristics of the interventions performed, instruments used to evaluate the outcomes, and results of the included studies. Two independent reviewers will perform the data extraction, and the disagreements will be resolved by a third reviewer. When data are not available in the manuscripts or in case of uncertainty, the authors may be contacted for clarification.

2.5 Assessment of Risk of Bias

Risk of bias will be assessed with the PEDro (Physiotherapy Evidence Database) scale, which has good levels of validity and reliability and is strongly correlated with the Cochrane risk of bias tool[23 24]. The PEDro scale evaluates the risk of bias and the statistical reporting of randomized controlled trials. This scale has 11 items: eight items (items 2-9) related to risk of bias (random allocation, concealed allocation, baseline comparability, blinded subjects,

blinded therapists, blinded assessors, adequate follow-up, and intention-to-treat analysis) and two items (10 and 11) related to statistical reporting (between-group comparisons and point estimates and variability)[24]. The first item (eligibility criteria) is not considered in the total score because it is related to external validity.

The total PEDro score ranges from 0 to 10 points, and the higher the score is, the better the article in terms of risk of bias and statistical reporting[24]. For trials that are not available in the PEDro database, the scale will be applied by two independent reviewers and a third reviewer will mediate any disagreement. Studies will be considered 'low risk' if they score 6 points or higher. Studies with scores below 6 will be considered 'high risk'[25].

2.6 Measures of treatment effect

The treatment effects for continuous data will be reported as mean difference with 95% confidence intervals. If the outcomes are evaluated by different scales, they will be converted to a common scale ranging from 0 to 100. The treatment effects of categorical outcomes will be calculated using the risk ratio (RR) with 95% confidence intervals.

When there are sufficiently homogeneous clinical trials in a comparison, a meta-analysis will be performed using the random effects model for the follow-up periods: short term (outcomes assessed closer to 4 weeks post-randomization); medium term (outcomes assessed closer to 6 months after randomization); and long term (outcomes assessed closer to 1 year after randomization)[22]. For the interpretation of minimal clinically important difference from the patient's point of view, we will consider 2 points on the Visual Analog Scale for Pain, ranging from 0 to 10, 5 points in the Roland Morris Disability Questionnaire, and 10 points in the Oswestry Disability Index in between-group comparisons.

2.7 Heterogeneity Analysis

The chi-square test will be used to identify the heterogeneity in the data from the studies. The magnitude of the heterogeneity will be confirmed by calculation of the I^2 statistic (range from 0 to 100%)[24]. An I^2 above 50% indicates significant heterogeneity and will result in a reduction of one level in the quality of the evidence due to inconsistency[22 25].

2.8 Synthesis of data

The quality of the evidence will be classified using the GRADE (Grading of Recommendations Assessment, Development and Evaluation) approach[25] and according to the Cochrane Handbook of Systematic Reviews[25]. The quality of evidence will be assessed for each comparison[26]. The quality of the evidence will be classified into four levels (high, moderate, low, and very low) and these levels represent our confidence in the estimated treatment effects[27] (Table 1).

Table 1. Level of quality of evidence (GRADE).

**Quality level**
**High quality**
• There are consistent findings among at least 75% of participants.<br>• Consistent low risk of bias, with accurate data and no known or suspected publication bias.<br>• Future studies are unlikely to change the estimated results.
**Moderate quality**
• There is moderate confidence in the estimated effect.<br>• Future studies are likely to have a significant impact on the confidence of the treatment effect estimation and may alter the treatment effect estimate.
**Low quality**

- Confidence in the treatment effect is limited.
- Additional research is very likely to have a significant impact on the confidence of the estimated treatment effect and it is likely to change the estimate.

Very low quality

- Confidence in the estimated treatment effect is very limited.
- Results are uncertain.

The initial classification of the quality of the evidence is defined according to the design of the studies. The randomized controlled trial is the most appropriate study design for questions related to intervention and its quality of the evidence starts as high according to the GRADE approach[28]. The quality of the evidence will be based on five factors, where for each factor not found, the quality of the evidence may be downgraded by one level (from high to moderate, low or very low). The five factors are:

- Methodological limitations (risk of bias): the quality of the evidence will be downgraded if there are methodological limitations that indicate a greater propensity for biases, thus reducing confidence in the estimation of the effect of the studies[28]. The evidence will be downgraded by one level when more than 25% of the studies included in a given comparison are classified as high risk of bias.

- Inconsistency: the quality of the evidence will be downgraded if significant heterogeneity is observed in the results, even after any sensitivity analysis of the hypotheses[29]. The evidence will be downgraded by one level when the inconsistency is greater than 50%.

- Indirect evidence: the quality of the evidence will be downgraded when participants, interventions, or outcomes from the assessed studies are essentially different from those considered in the research question or the clinical guideline or when there are no direct

comparisons between the interventions[30]. The evidence will be downgraded by one level when more than 50% of the participants are not related to the target audience of the study.

- Imprecision: the main criterion used by the GRADE system to define the accuracy of the estimates is the 95% confidence interval[30]. The evidence will be downgraded by level when there are less than 400 participants in the comparison for continuous outcomes and fewer than 300 participants for categorical outcomes.

- Publication bias: The funnel plot[31] used for meta-analyses with ten studies or more will be used to verify publication bias. Studies with low accuracy and with small samples will be distributed symmetrically in the widest part of the funnel and studies with higher accuracy and larger sample sizes will be closer to the actual result and located in the narrowest part of the funnel[31].

3. DISCUSSION

This systematic review aims to provide the best available evidence on the effectiveness of aerobic exercise in patients with chronic nonspecific low back pain on pain and disability outcomes. All recommendations of the Cochrane Handbook of Systematic Reviews will be followed for the review to be of high quality. We believe that the findings of this systematic review will be important because aerobic exercise is affordable, cost effective, and commonly used by the general population. To date, we are not aware of any systematic reviews that have attempted to investigate the efficacy of aerobic exercise alone compared to other therapies in reducing the pain and disability of patients with chronic nonspecific low back pain. Therefore, this evidence will inform healthcare providers and patients about the potential benefits of this intervention. Furthermore, this review has the potential to identify gaps in the literature that could be addressed in future studies.

3.1 Ethics and Dissemination

The results of the study will be part of a doctoral thesis and will be published in peer-reviewed international journals and presented in international conferences.

3.2 Contribution from authors:

I.D.S. and L.O.P.C. developed the research questions, methods section and wrote the first draft of the manuscript. M.A.O., A.C.L and N.T.B.O. contributed with the development of methods, search strategies and writing this manuscript. All authors contributed to the drafting of the review protocol and approved the final manuscript.

Conflict of Interest: none declared.

Funding: IDS received a PhD scholarship from FAPEAM (Fundação de Amparo à Pesquisa do Amazonas – Brazil).

REFERENCES

1. Vos T, Allen C, Arora M, et al. Global, regional, and national incidence, prevalence, and years lived with disability for 310 diseases and injuries, 1990-2015: a systematic analysis for the Global Burden of Disease Study 2015. *Lancet* 2016;388(10053):1545.
2. Maher C, Underwood M, Buchbinder R. Non-specific low back pain. *Lancet* 2017;389(10070):736-47.
3. Delitto A, George SZ, Van Dillen L, et al. Low Back Pain. Clinical Practice Guidelines Linked to the International Classification of Functioning, Disability, and Health from the Orthopaedic Section of the American Physical Therapy Association. *J Orthop Sports Phys Ther* 2012;42(4):A1-57.
4. Indrakanti SS, Weber MH, Takemoto SK, et al. Value-based care in the management of spinal disorders: a systematic review of cost-utility analysis. *Clin Orthop Relat Res®* 2012;470(4):1106-23.
5. Costa LdCM, Maher CG, McAuley JH, et al. Prognosis for patients with chronic low back pain: inception cohort study. *BMJ* 2009;339:b3829.
6. Chou R, Qaseem A, Snow V, et al. Diagnosis and treatment of low back pain: a joint clinical practice guideline from the American College of Physicians and the American Pain Society. *Ann Intern Med* 2007;147(7):478-91. [published Online First: 2007/10/03]
7. Stochkendahl MJ, Kjaer P, Hartvigsen J, et al. National Clinical Guidelines for non-surgical treatment of patients with recent onset low back pain or lumbar radiculopathy. *Eur Spine J* 2017:1-16.
8. NNational GCU. Low Back Pain and Sciatica in Over 16s: Assessment and Management. 2016
9. Schreuder L, Peters G, Nijhuis-van der Sanden R, et al. Aerobic exercise in children with oxidative phosphorylation defects. *Neurol Int* 2010;2(1):4.
10. Hildebrandt J, Ursin H, Mannion AF, et al. European guidelines for the management of chronic non-specific low back pain. *Norway: European Commission, Research Directorate-General, Department of Policy, Co-ordination and Strategy* 2004
11. van der Velde G, Mierau D. The effect of exercise on percentile rank aerobic capacity, pain, and self-rated disability in patients with chronic low-back pain: a retrospective chart review. *Arch Phys Med Rehabil* 2000;81(11):1457-63.
12. Verbunt JA, Seelen HA, Vlaeyen JW, et al. Disuse and deconditioning in chronic low back pain: concepts and hypotheses on contributing mechanisms. *Eur J Pain* 2003;7(1):9-21.
13. Stoppler MC, Shiel W. Endorphins: Natural pain and stress fighters: Diperoleh tanggal, 2014.
14. Wertli MM, Rasmussen-Barr E, Held U, et al. Fear-avoidance beliefs—a moderator of treatment efficacy in patients with low back pain: a systematic review. *Spine J* 2014;14(11):2658-78.
15. Hayden JA, van Tulder MW, Malmivaara A, et al. Exercise therapy for treatment of non-specific low back pain. *Cochrane Database Syst Rev* 2005(3):Cd000335. [published Online First: 2005/07/22]
16. Meng X-G, Yue S-W. Efficacy of aerobic exercise for treatment of chronic Low back pain: a meta-analysis. *Am J Phys Med Rehabil* 2015;94(5):358-65.
17. A systematic review of the effects of exercise and physical activity on non-specific chronic low back pain. Healthcare; 2016. Multidisciplinary Digital Publishing Institute.
18. Mancini MC, Cardoso JR, Sampaio RF, et al. Tutorial for writing systematic reviews for the Brazilian Journal of Physical Therapy (BJPT). *Braz J Phys Ther* 2014;18(6):471-80.
19. Chiarotto A, Deyo RA, Terwee CB, et al. Core outcome domains for clinical trials in non-specific low back pain. *Eur Spine J* 2015;24(6):1127-42.
20. Chiarotto A, Boers M, Deyo RA, et al. Core outcome measurement instruments for clinical trials in nonspecific low back pain. *Pain* 2018;159(3):481.
21. Shiwa SR, Moseley AM, Maher CG, et al. Language of publication has a small influence on the quality of reports of controlled trials of physiotherapy interventions. *J Clin Epidemiol* 2013;66(1):78-84.

22. Furlan AD, Malmivaara A, Chou R, et al. 2015 updated method guideline for systematic reviews in the Cochrane Back and Neck Group. *Spine* 2015;40(21):1660-73.
23. Maher CG, Sherrington C, Herbert RD, et al. Reliability of the PEDro scale for rating quality of randomized controlled trials. *Phys Ther* 2003;83(8):713.
24. Yamato TP, Maher C, Koes B, et al. The PEDro scale had acceptably high convergent validity, construct validity, and interrater reliability in evaluating methodological quality of pharmaceutical trials. *J Clin Epidemiol* 2017
25. Higgins J, Green Se. Cochrane handbook for systematic reviews of interventions Version 5.1.0 (updated March2011): The Cochrane Collaboration, 2011. Available from http://handbook.cochrane.org.
26. Guyatt GH, Oxman AD, Vist GE, et al. GRADE: an emerging consensus on rating quality of evidence and strength of recommendations. *BMJ* 2008;336(7650):924.
27. Balshem H, Helfand M, Schünemann HJ, et al. GRADE guidelines: 3. Rating the quality of evidence. *J Clin Epidemiol* 2011;64(4):401-06.
28. Guyatt GH, Oxman AD, Vist G, et al. GRADE guidelines: 4. Rating the quality of evidence—study limitations (risk of bias). *J Clin Epidemiol* 2011;64(4):407-15.
29. Guyatt GH, Oxman AD, Kunz R, et al. GRADE guidelines: 7. Rating the quality of evidence—inconsistency. *J Clin Epidemiol* 2011;64(12):1294-302.
30. Guyatt GH, Oxman AD, Kunz R, et al. GRADE guidelines 6. Rating the quality of evidence—imprecision. *J Clin Epidemiol* 2011;64(12):1283-93.
31. Sterne JA, Egger M. Funnel plots for detecting bias in meta-analysis: guidelines on choice of axis. *J Clin Epidemiol* 2001;54(10):1046-55.

Additional file 1

Search Strategy for MEDLINE (OVID):

Part A: Generic search for randomized controlled trials and controlled clinical trials

1. randomized controlled trial.pt.
2. controlled clinical trial.pt.
3. comparative study.pt.
4. clinical trial.pt.
5. randomized.ab.
6. placebo.ab,ti.
7. drug therapy.fs.
8. randomly.ab,ti.
9. trial.ab,ti.
10. groups.ab,ti.
11. or/1-10
12. (animals not (humans and animals)).sh.
13. 11 not 12

Part B: Specific search for thoracic, low back, sacrum, and coccyx problems

14. dorsalgia.ti,ab.
15. exp Back Pain/
16. backache.ti,ab.
17. exp Low Back Pain/
18. (lumbar adj pain).ti,ab.
19. coccyx.ti,ab.
20. coccydynia.ti,ab.
21. sciatica.ti,ab.
22. sciatic neuropathy/
23. spondylosis.ti,ab.
24. lumbago.ti,ab.
25. back disorder$.ti,ab.
26. or/14-25

Part C: Specific search for Exercise   Abbreviation: ti (title) ab (abstract)

27. physical activity.ti,ab
28. swimming.ti,ab
29. running.ti,ab
30. walking.ti,ab
31. rowing.ti,ab
32. weight.ti,ab
33. pedal.ti,ab
34. bicycling.ti,ab
35. cycling.ti,ab
36. jogging.ti,ab
37. skating.ti,ab
38. dancing.ti,ab
39. strengthening.ti,ab
40. gymnasti.ti,ab
41. golf.ti,ab
42. mountaineer.ti,ab
43. physical recreation.ti,ab
44. exercises.ti,ab
45. exercise tolerance.ti,ab
46. aerobic capacity.ti,ab
47. physical capacity.ti,ab
48. physical endurance.ti,ab
49. physical near endurance.ti,ab
50. leisure activit.ti,ab
51. physical activity.ti,ab
52. activities physical.ti,ab
53. activity physical.it,ab
54. physical activities.it,ab
55. exercise physical.it,ab
56. exercises physical.it,ab
57. physical exercise.it,ab
58. physical exercises.it,ab

59. acute exercise.it,ab
60. acute exercises.it,ab
61. exercise acute.it,ab
62. exercises acute.it,ab
63. exercise isometric.it,ab
64. exercises isometric.it,ab
65. isometric exercises.it,ab
66. isometric exercise.it,ab
67. exercise aerobic.it,ab
68. aerobic exercise.it,ab
69. aerobic exercises.it,ab
70. exercises aerobic.it,ab
71. exercise training.it,ab
72. exercise trainings.it,ab
73. training exercise.it,ab
74. trainings exercise.it,ab
75. therapy exercise.it,ab
76. exercise therapies.it,ab
77. therapies exercise.it,ab
78. rehabilitation exercise.it,ab
79. exercise rehabilitation.it,ab
80. exercises rehabilitation.it,ab
81. rehabilitation exercises.it,ab
82. remedial exercise.it,ab
83. exercise remedial.it,ab
84. exercises remedial.it,ab
85. remedial exercise.it,ab
86. movemen techniques exercise.it,ab
87. exercise movement technics.it,ab
88. exertion physical.it,ab
89. exertions physical.it,ab
90. physical exertions.it,ab
91. physical effort.it,ab
92. effort physica.it,ab

93. efforts physical.it,ab

94. physical efforts.it,ab

95. cardiorespiratory fitness.it,ab

96. sport.it,ab

97. athletics.it,ab

98. athletic.it,ab

99. aquarobics.it,ab

100. dance workout.it,ab

101. drill.it,ab

102. high impact.it,ab

103. low Impact.it,ab

104. slimnastics.it,ab

105. step.it,ab

106. warm-up.it,ab

107. workout.it,ab

108. endurance.it,ab

109. or/27-109

Results (all RCTs and CCTs for spinal disorders and aerobic exercise)

110. 26 or 109

111. 13 and 110

Search strategy for EMBASE (Ovid)

Part A: Generic search for randomized controlled trials and controlled clinical trials

1 Clinical Article/

2 exp Clinical Study/

3 Clinical Trial/

4 Controlled Study/

5 Randomized Controlled Trial/

6 Major Clinical Study/

7 Double Blind Procedure/

8 Multicenter Study/

9 Single Blind Procedure/

10 Phase 3 Clinical Trial/

11 Phase 4 Clinical Trial/

12 crossover procedure/

13 placebo/

14 or/1-13

15 allocat$.mp.

16 assign$.mp.

17 blind$.mp.

18 (clinic$ adj25 (study or trial)).mp.

19 compar$.mp.

20 control$.mp.

21 cross?over.mp.

22 factorial$.mp.

23 follow?up.mp.

24 placebo$.mp.

25 prospectiv$.mp.

26 random$.mp.

27 ((singl$ or doubl$ or trebl$ or tripl$) adj25 (blind$ or mask$)).mp.

28 trial.mp.

29 (versus or vs).mp.

30 or/15-29

31 14 OR 30

32 exp animals/ or exp invertebrate/ or animal experiment/ or animal model/ or animal tissue/ or animal cell/ or nonhuman/

33 human/ or normal human/ or human cell/

34 32 and 33

35 32 not 34

36 31 not 35

Part B: Specific search for thoracic, low back, sacrum, and coccyx problems

37 dorsalgia.mp.

38 back pain.mp.

39 exp LOW BACK PAIN/

40 exp BACKACHE/

41 (lumbar adj pain).mp.

42 coccyx.mp.

43 coccydynia.mp.

44 sciatica.mp.

45 exp ISCHIALGIA/

46 spondylosis.mp.

47 lumbago.mp.

48 back disorder$.ti,ab.

49 or/37-48

Part C: Specific search for Exercise

50. physical activity.mp.
51. swimming.mp.
52. running.mp.
53. walking.mp.
54. rowing.mp.
55. weight.mp.
56. pedal.mp.
57. bicycling.mp.
58. cycling.mp.
59. jogging.mp.
60. skating.mp.
61. dancing.mp
62. strengthening.mp.
63. gymnasti.mp.
64. golf.mp.
65. mountaineer.mp.
66. physical recreation.mp.
67. exercise$.mp.
68. exercise tolerance.mp.
69. aerobic capacity.mp.
70. physical capacity.mp.

71. physical endurance.mp.
72. physical near endurance.mp.
73. leisure activit.mp.
74. physical activity.mp.
75. activities physical.mp.
76. activity physical.mp.
77. physical activities.mp.
78. exercise physical.mp.
79. exercises physical.mp.
80. physical exercise.mp.
81. physical exercises.mp.
82. acute exercise.mp.
83. acute exercises.mp.
84. exercise acute.mp.
85. exercises acute.mp.
86. exercise isometric.mp.
87. exercises isometric.mp.
88. isometric exercises.mp
89. exercise aerobic.it,ab
90. aerobic exercise$.mp.
91. exercises aerobic.mp.
92. exercise training$.mp.
93. training$ exercise$.mp.
94. therapy exercise.mp
95. exercise therapies.mp.
96. therapies exercise.mp.
97. rehabilitation exercise.mp.
98. exercise rehabilitation.mp.
99. exercises rehabilitation.mp.
100. rehabilitation exercises.mp.
101. remedial exercise.mp.
102. exercise remedial.mp.
103. exercises remedial.mp
104. remedial exercise.mp.

105. movemen techniques exercise.mp.

106. exercise movement technics.mp.

107. exertion physical.mp.

108. exertions physical.mp.

109. physical exertions.mp.

110. physical effort.mp.

111. effort physica.mp.

112. efforts physical.mp.

113. physical efforts.mp.

114. cardiorespiratory fitness.mp.

115. sport.mp.

116. athletics.mp.

117. athletic.mp.

118. aquarobics.mp.

119. dance workout.mp.

120. drill.mp.

121. high impact.mp.

122. low Impact.mp.

123. slimnastics.mp.

124. step.mp.

125. warm-up.mp.

126. workout.mp.

127. endurance.mp.

128. or/50-128

Results (all RCTs and CCTs for spinal disorders and aerobic exercise)

129. 49 or 128

130. 36 and 129

Search strategy for CINAHL (EBSCO)

Part A: Generic search for randomized controlled trials and controlled clinical trials

S28 S26 NOT S27

S27 (MH "Animals")

S26 S7 or S12 or S19 or S25

S25 S20 or S21 or S22 or S23 or S24

S24 volunteer*

S23 prospectiv*

S22 control*

S21 followup stud*

S20 follow-up stud*

S19 S13 or S14 or S15 or S16 or S17 or S18

S18 (MH "Prospective Studies+")

S17 (MH "Evaluation Research+")

S16 (MH "Comparative Studies")

S15 latin square

S14 (MH "Study Design+")

S13 (MH "Random Sample")

S12 S8 or S9 or S10 or S11

S11 random*

S10 placebo*

S9 (MH "Placebos")

S8 (MH "Placebo Effect")

S7 S1 or S2 or S3 or S4 or S5 or S6

S6 triple-blind

S5 single-blind

S4 double-blind

S3 clinical W3 trial

S2 "randomi?ed controlled trial*"

S1 (MH "Clinical Trials+")

Part B: Specific search for thoracic, low back, sacrum, and coccyx problems

S48 S35 or S43 or S47

S47 S44 or S45 or S46

S46 "lumbago"

S45 (MH "Spondylolisthesis") OR (MH "Spondylolysis")

S44 (MH "Thoracic Vertebrae")

S43 S36 or S37 or S38 or S39 or S40 or S41 or S42

S42 lumbar N2 vertebra

S41 (MH "Lumbar Vertebrae")

S40 "coccydynia" OR "back disorder*"

S39 "coccyx"

S38 "sciatica"

S37 (MH "Sciatica")

S36 (MH "Coccyx")

S35 S29 or S30 or S31 or S32 or S33 or S34

S34 lumbar N5 pain

S33 lumbar W1 pain

S32 "backache"

S31 (MH "Low Back Pain")

S30 (MH "Back Pain+")

S29 "dorsalgia"

Part C: Specific search for Exercise

S143 S57 or S64 or S71 or S78 or S85 or S92 or S99 or S107 or S115 or S122 or S129 or S136 or S142

S142 S137 or S138 or S139 or S140 or S141

S141 (MH “physical activity+”)

S140 (MH “swimming”)

S139 (MH “running+”)

S138 (MH “walking+”)

S137 (MH “rowing”)

S136 S130 or S131 or S132 or S133 or S134 or S135

S135 (MH “weight”)

S134 (MH “pedal+”)

S133 (MH “bicycling+”)

S132 (MH “cycling+”)

S131 (MH “jogging+”)

S130 (MH “skating”)

S129 S123 or S124 or S125 or S126 or S127 or S128

S128 (MH “dancing”)

S127 (MH “strengthening”)

S126 (MH “gymnasti”)

S125 (MH “golf”)

S124 (MH “mountainee”)

S123 (MH “physical recreation+”)

S122 S116 or S117 or S118 or S119 or S120 or S121

S121 (MH “exercises+”)

S120 (MH “exercise tolerance+”)

S119 (MH “aerobic capacity+”)

S118 (MH “physical capacity+”)

S117 (MH “physical endurance+”)

S116 (MH “physical near endurance”)

S115 S108 or S109 or S110 or S111 or S112 or S113 or S114

S114 (MH “leisure Activit”)

S113(MH “physical Activity+”)

S112 (MH “activities physical+”)

S111 (MH “activity physica+”)

S110 (MH “physical activities+”)

S109 (MH “exercise physical+”)

S108(MH “exercises physical+”)

S107 S100 or S101 or S102 or S103 or S104 or S105 or S106

S106 (MH “physical exercise+”)

S105 (MH “physical exercises+”)

S104 (MH “acute exercise+”)

S103 (MH “acute exercises+”)

S102 (MH “exercise acute+”)

S101 (MH “exercises acute+”)

S100 (MH “exercise isometric”)

S99 S93 or S94 or S95 or S96 or S97 or S98

S98 (MH “exercises isometric”)

S97(MH “isometric exercises”)

S96 (MHI “sometric exercise”)

S95 (MH “exercise aerobic+”)

S94 (MH “aerobic exercise+”)

S93 (MH “aerobic exercises+”)

S92 S86 or S87 or S89 or S90 or S91 or S92

S91 (MH “exercise training+”)

S90 (MH “exercise trainings+”)

S89 (MH “training exercise+”)

S88 (MH “trainings exercise+”)

S87 (MH “therapy exercise+”)

S86 (MH “exercise therapies+”)

S85 S79 or S80 or S81 or S82 or S83 or S84

S84(MH “therapies exercise+”)

S83 (MH “rehabilitation exercise+”)

S82 (MH “exercise rehabilitation+”)

S81 (MH “exercises rehabilitation+”)

S80 (MH “rehabilitation exercises+)

S79 (MH “remedial exercise+”)

S78 S72 or S73 or S74 or S75 or S76 or S77

S77 (MH “exercise remedial+”)

S76 (MH “exercises remedial+”)

S75 (MH “remedial exercise+”)

S74 (MH “movement techniques exercise+”)

S73 (MH “exercise movement technics+”)

S72 (MH “exertion physical)

S71 S65 or S66 or S67 or S68 or S69 or S70

S70 (MH “exertions physical”)

S69 (MH “physical exertions”)

S68 (MH “physical effort”)

S67 (MH “effort physical”)

S66 (MH “efforts physical”)

S65 (MH “physical efforts”)

S64 S58 or S59 or S60 or S61 or S62 or S63

S63 (MH “cardiorespiratory fitness+”)

S62(MH “sport+”)

S61 (MH “athletics”)

S60 (MH “athletic”)

S59 (MH “aquarobics”)

S58(MH “dance workout”)

S57 S49 or S50 or S51 or S52 or S53 or S54 or S55 or S56

S56 (MH “drill”)

S55 (MH “high impact”)

S54 (MH “low impact”)

S53 (MH “slimnastics”)

S52 (MH “step”)

S51 (MH “warm-up”)

S50 (MH “workout”)

S49 (MH “endurance”)

Results (all RCTs and CCTs for spinal disorders e exercise aerobic)

S144 S143 or S48

Search strategy for CENTRAL – online (Cochrane Library)

Part A: Specific search for back pain and spinal disorders

#1 MeSH descriptor Back Pain explode all trees

#2 dorsalgia

#3 backache

#4 MeSH descriptor Low Back Pain explode all trees

#5 (lumbar next pain) or (coccyx) or (coccydynia) or (spondylosis)

#6 MeSH descriptor Spine explode all trees

#7 MeSH descriptor Spinal Diseases explode all trees

#8 (lumbago) or (discitis) or (disc near degeneration) or (disc near prolapse) or (disc near herniation)

#9 spinal fusion

#10 spinal neoplasms

#11 facet near joints

#12 MeSH descriptor Intervertebral Disk explode all trees

#13 postlaminectomy

#14 arachnoiditis

#15 failed near back

#16 MeSH descriptor Cauda Equina explode all trees

#17 lumbar near vertebra*

#18 spinal near stenosis

#19 slipped near (disc* or disk*)

#20 degenerat* near (disc* or disk*)

#21 stenosis near (spine or root or spinal)

#22 displace* near (disc* or disk*)

#23 prolap* near (disc* or disk*)

#24 MeSH descriptor Sciatic Neuropathy explode all trees

#25 sciatic*

#26 back disorder*

#27 back near pain

#28 (#1 OR #2 OR #3 OR #4 OR #5 OR #6 OR #7 OR #8 OR #9 OR #10 OR #11 OR #12 OR #13 OR #14 OR #15 OR #16 OR #17 OR #18 OR #19 OR #20 OR #21 OR #22 OR #23 OR #24 OR #25 OR #26 OR #27)

Part B: Specific search for Exercise

#29 MeSH Physical Activity explode all trees

#30 MeSH Swimming explode all trees

#31 MeSH Running explode all trees

#32 MeSH Walking explode all trees

#33 rowing

#34 weight

#35 pedal

#36 MeSH Bicycling explode all trees

#37 MeSH Cycling explode all trees

#38 MeSH Jogging explode all trees

#39 skating

#40 dancing

#41 strengthening

#42 gymnasti

#43 golf

#44 mountainee

#45 MeSH Physical Recreation explode all trees

#46 MeSH Exercises explode all trees

#47 MeSH Exercise tolerance explode all trees

#48 MeSH Aerobic capacity explode all trees

#49 MeSH Physical capacity explode all trees

#50 MeSH Physical endurance explode all trees

#51 physical near endurance

#52 leisure activit

#53 MeSH Physical Activity explode all trees

#54 MeSH Activities, Physical explode all trees

#55 MeSH Activity, Physical explode all trees

#56 MeSH Physical Activities explode all trees

#57 MeSH Exercise, Physical explode all trees

#58 MeSH Exercises, Physical explode all trees

#59 MeSH Physical Exercise explode all trees

#60 MeSH Physical Exercises explode all trees

#61 acute exercise*

#62 acute exercises*

#63 exercise acute*

#64 exercises acute*

#65 exercise isometric

#66 exercises isometric

#67 isometric exercises

#68 isometric exercise

#69 MeSH Exercise, Aerobic explode all trees

#70 MeSH Aerobic Exercise explode all trees

#71 MeSH Aerobic Exercises explode all trees

#72 MeSH Exercises, Aerobic explode all trees

#73 MeSH Exercise Training explode all trees

#74 MeSH Trainings Exercise explode all trees

#75 MeSH Therapy Exercise explode all trees

#76 MeSH Exercise Therapies explode all trees
#77 therapies exercise*
#78 MeSH Rehabilitation Exercise explode all trees
#79 MeSH Exercise Rehabilitation explode all trees
#80 MeSH Exercises Rehabilitation explode all trees
#81 MeSH Rehabilitation Exercises explode all trees
#82 remedial exercise
#83 exercise remedial
#84 exercises remedial
#85 remedial exercise
#86 movement techniques exercise*
#87 exercise movement technics*
#88 exertion physical
#89 exertions physical
#90 physical exertions*
#91 physical effort
#92 effort physical
#93 efforts physical
#94 physical efforts
#95 MeSH Cardiorespiratory Fitness explode all trees
#96 MeSH Sport explode all trees
#97 MeSH Athletics explode all trees
#98 MeSH Athletic explode all trees
#99 MeSH Aquarobics explode all trees
#100 dance workout
#101 drill
#102 high impact
#103 MeSH Low Impact explode all trees
#104 slimnastics
#105 step
#106 MeSH Warm-up explode all trees
#107 MeSH Workout explode all trees
#108 MeSH Endurance explode all trees

#109 (#29 OR #30 OR #31 OR #32 OR #33 OR #34 OR #35 OR #36 OR #37 OR #38 OR #39 OR #40 OR #41 OR #42 OR #43 OR #44 OR #45 OR #46 OR #47 OR #48 OR #49 OR #50 OR #51 OR #52 OR #53 OR #54 OR #55 OR #56 OR #57 OR #58 OR #59 OR #60 OR #61 OR #62 OR #63 OR #64 OR #65 OR #66 OR #67 OR #68 OR #69 OR #70 OR #71 OR #72 OR #73 OR #74 OR #75 OR #76 OR #77 OR #78 OR #79 OR #80 OR #81 OR#82 OR #83 OR #84 OR #85 OR #86 OR #87 OR #88 OR #89 OR #90 OR #91 OR #92 OR #93 OR #94 OR #95 OR #96 OR #97 OR #98 OR #99 OR #100 OR #101 OR #102 OR #103 OR #104 OR #105 OR #106 OR #107 OR #108

Results (all RCTs and CCTs for spinal disorders)

#40 (#28 OR #39)

Search strategy for PsycInfo (OVID)

Part A: RCT filter

1. clinical trials/
2. controlled trial.mp.
3. RCT.mp.
4. (Random* adj3 trial).mp.
5. (clin* adj3 trial).mp.
6. (sing* adj2 blind*).mp.
7. (doub* adj2 blind*).mp.
8. placebo.mp. or exp Placebo/
9. latin square.mp.
10. (random* adj2 assign*).mp.
11. prospective studies/
12. (prospective adj stud*).mp.
13. (comparative adj stud*).mp.
14. treatment effectiveness evaluation/
15. treatment effectiveness evaluation/
16. (evaluation adj stud*).mp.
17. exp Posttreatment Followup/
18. followup stud*.mp.

19. or/1-18

Part B: back pain, spinal disorders

20. back pain/

21. lumbar spinal cord/

22. (low adj back adj pain).mp. [mp=title, abstract, subject headings, heading word, drug trade name, original title, device manufacturer, drug manufacturer, device trade name, keyword]

23. (back adj pain).mp. [mp=title, abstract, subject headings, heading word, drug trade name, original title, device manufacturer, drug manufacturer, device trade name, keyword]

24. spinal column/

25. (lumbar adj2 vertebra*).mp. [mp=title, abstract, subject headings, heading word, drug trade name, original title, device manufacturer, drug manufacturer, device trade name, keyword]

26. coccyx.mp.

27. sciatica.mp.

28. lumbago.mp.

29. dorsalgia.mp.

30. back disorder*.mp.

31. ((disc or disk) adj degenerat*).mp. [mp=title, abstract, subject headings, **heading word, drug trade name, original title, device manufacturer, drug** manufacturer, device trade name, keyword]

32. ((disc or disk) adj herniat*).mp.

33. ((disc or disk) adj prolapse*).mp.

34. (failed adj back).mp.

35. or/20-35

results

41. 19 and 40

Part C: Specific search for Exercise

36. physical activity.mp.

37. swimming.mp.

38. running.mp.
39. walking.mp.
40. rowing.mp.
41. weight.mp.
42. pedal.mp.
43. bicycling.mp.
44. cycling.mp.
45. jogging.mp.
46. skating.mp.
47. dancing.mp
48. strengthening.mp.
49. gymnasti.mp.
50. golf.mp.
51. mountaineer.mp.
52. physical recreation.mp.
53. exercise$.mp.
54. exercises tolerance.mp.
55. aerobic capacity.mp.
56. physical capacity.mp.
57. physical endurance.mp.
58. physical near endurance.mp.
59. leisure activit.mp.
60. physical activity.mp.
61. activities physical.mp.
62. activity physical.mp.
63. physical activities.mp.
64. exercise physical.mp.
65. exercises physical.mp.
66. physical exercise.mp.
67. physical exercises.mp.
68. acute exercise.mp.
69. acute exercises.mp.
70. exercise acute.mp.
71. exercises acute.mp.

72. exercise isometric.mp.
73. exercises isometric.mp.
74. isometric exercises.mp
75. exercise aerobic.it,ab
76. aerobic exercise$.mp.
77. exercises aerobic.mp.
78. exercise training$.mp.
79. training$ exercise$.mp.
80. therapy exercise.mp
81. exercise therapies.mp.
82. therapies exercise.mp.
83. rehabilitation exercise.mp.
84. exercise rehabilitation.mp.
85. exercises rehabilitation.mp.
86. rehabilitation exercises.mp.
87. remedial exercise.mp.
88. exercise remedial.mp.
89. exercises remedial.mp
90. remedial exercise.mp.
91. movemen techniques exercise.mp.
92. exercise movement technics.mp.
93. exertion physical.mp.
94. exertions physical.mp.
95. physical exertions.mp.
96. physical effort.mp.
97. effort physica.mp.
98. efforts physical.mp.
99. physical efforts.mp.
100. cardiorespiratory fitness.mp.
101. sport.mp.
102. athletics.mp.
103. athletic.mp.
104. aquarobics.mp.
105. dance workout.mp.

106. drill.mp.
107. high impact.mp.
108. low Impact.mp.
109. slimnastics.mp.
110. step.mp.
111. warm-up.mp.
112. workout.mp.
113. endurance.mp.
114. or/36-114
